# Artificial intelligence in neurodegenerative diseases research: a bibliometric analysis since 2000

**DOI:** 10.3389/fneur.2025.1607924

**Published:** 2025-07-16

**Authors:** Yabin Zhang, Lei Yu, Yuting Lv, Tiantian Yang, Qi Guo

**Affiliations:** ^1^Department of Special Services, The 960th Hospital of the PLA Joint Logistics Support Force, Jinan, Shandong, China; ^2^Campus Clinic, Shandong University of Traditional Chinese Medicine, Jinan, Shandong, China; ^3^Department of Traditional Chinese Medicine, Shandong Provincial Hospital Affiliated to Shandong First Medical University, Jinan, Shandong, China

**Keywords:** artificial intelligence, neurodegenerative diseases, bibliometric, VOSviewer, CiteSpace, bibliometrix R

## Abstract

This bibliometric review examines the evolving landscape of artificial intelligence (AI) in neurodegenerative diseases research from 2000 to March 16, 2025, utilizing data from 1,402 publications (1,159 articles, 243 reviews) indexed in the Web of Science Core Collection. Through advanced tools - VOSviewer, CiteSpace, and Bibliometrix R - the study maps collaboration networks, keyword trends, and knowledge trajectories. Results reveal exponential growth post-2017, driven by advancements in deep learning and multimodal data integration. The United States (25.96%) and China (24.11%) dominate publication volume, while the UK exhibits the highest collaboration centrality (0.24) and average citations per publication (31.68). Core journals like *Scientific Reports* and *Frontiers in Aging Neuroscience* published the most articles in this field. Highly cited publications and burst references highlight important milestones in the development history. High-frequency keywords include “alzheimer’s disease,” “parkinson’s disease,” “magnetic resonance imaging,” “convolutional neural network,” “biomarkers,” “dementia,” “classification,” “mild cognitive impairment,” “neuroimaging,” and “feature extraction.” Key hotspots include intelligent neuroimaging analysis, machine learning methodological iterations, molecular mechanisms and drug discovery, and clinical decision support systems for early diagnosis. Future priorities encompass advanced deep learning architectures, multi-omics integration, explainable AI systems, digital biomarker-based early detection, and transformative technologies including transformers and telemedicine. This analysis delineates AI’s transformative role in optimizing diagnostics and accelerating therapeutic innovation, while advocating for enhanced interdisciplinary collaboration to bridge computational advances with clinical translation.

## 1 Introduction

Neurodegenerative diseases, such as Alzheimer’s disease and Parkinson’s disease, represent a significant and growing global health challenge, particularly as populations age. These diseases are characterized by progressive neural dysfunction, leading to cognitive impairments and motor dysfunctions. The increasing prevalence of these conditions underscores the urgent need for innovative therapeutic strategies to combat their debilitating effects (1). Concurrently, artificial intelligence (AI) has demonstrated transformative potential in medical research through deep learning models for neuroimaging segmentation (2), predictive algorithms for drug-target interactions (3), and multimodal frameworks integrating genetic and clinical data (4). However, the integration of AI in neurodegenerative diseases research faces inherent complexities from interdisciplinary convergence (5). This challenge requires synergistic expertise in neurobiology, computational modeling, and clinical validation (6). Medical researchers, computer scientists, and bioinformaticians often operate within siloed frameworks, leading to fragmented methodologies. Consequently, macro-level evaluations are required to delineate global collaboration patterns, knowledge architecture shifts, and emergent technological trajectories in this interdisciplinary landscape. A systematic analysis of these dimensions, however, remains conspicuously underexplored in existing scholarships.

Bibliometric analysis serves as a powerful tool to map research landscapes by revealing co-authorship networks among countries/regions, institutions, and authors, keyword co-occurrence, and assessing journal influence (7, 8). It integrates multidimensional data - including authorship, citations, and keyword co-occurrence — with visualization techniques to generate scientific mapping that delineates the structure and dynamics of domain development (9). This approach helps identify emerging research frontiers and inform resource allocation strategies, while fostering interdisciplinary synergies and translational medical advancements. By systematically tracking knowledge trajectories and innovation patterns, it enables researchers to decode field dynamics, optimize scientific decision-making, and bridge disciplinary divides. Importantly, this study represents the first bibliometric analysis of AI applications in neurodegenerative diseases research since 2000.

## 2 Materials and methods

### 2.1 Data source and search strategy

**Figure 1:**
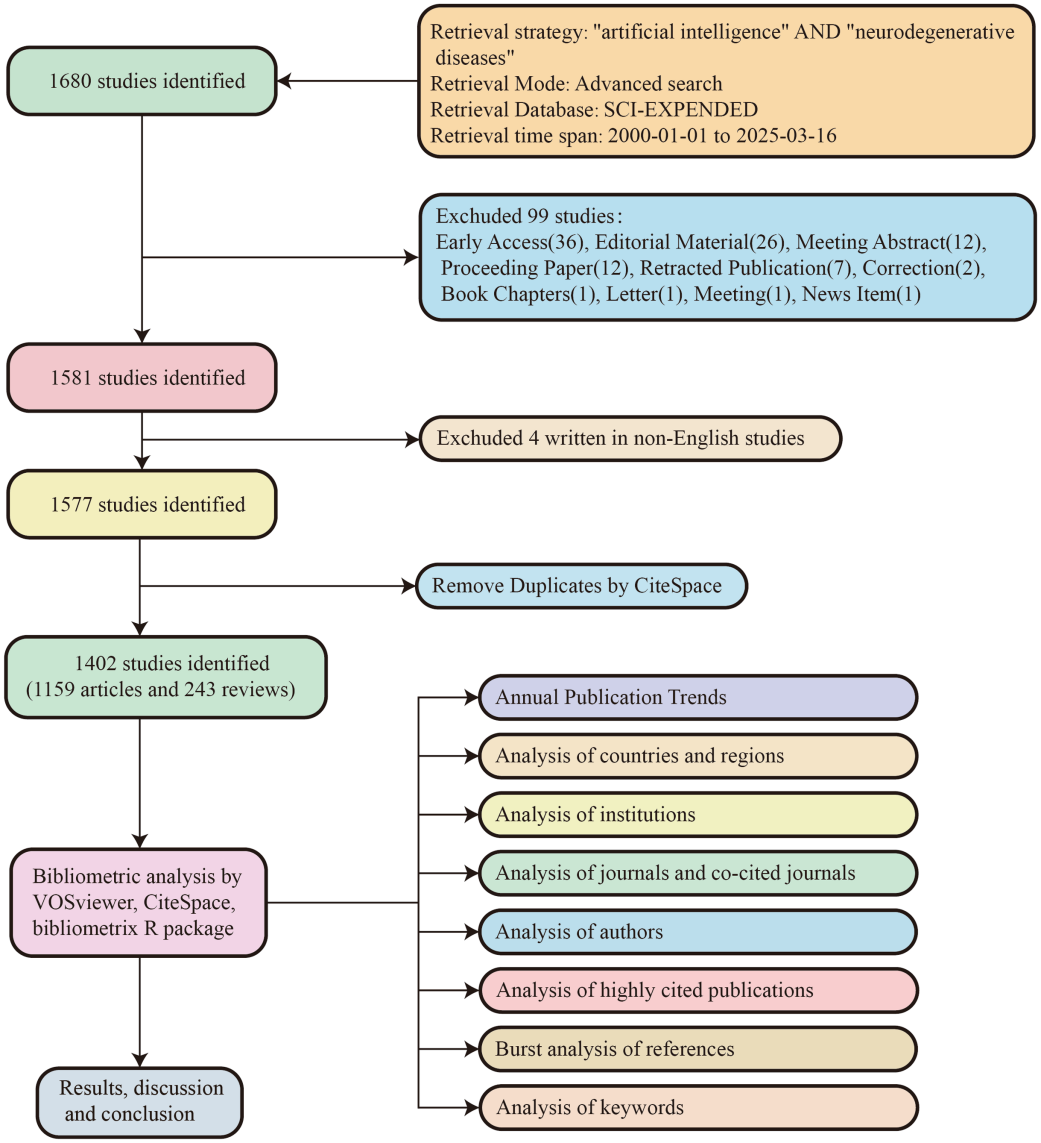
Flow chart of literature search and bibliometric analysis.

As shown in Figure 1, we conducted a comprehensive literature search in the Web of Science Core Collection (WoSCC) database. The search strategy employed the retrieval formula: (TS = (“Neurodegenerative Diseases” OR “Neurodegenerative Disease” OR “Degenerative Neurologic Disorders” OR “Degenerative Neurologic Disorder” OR “Neurologic Disorder, Degenerative” OR “Neurologic Disorders, Degenerative” OR “Nervous System Degenerative Diseases” OR “Neurodegenerative Disorders” OR “Neurodegenerative Disorder” OR “Degenerative Diseases, Nervous System” OR “Degenerative Diseases, Neurologic” OR “Neurologic Degenerative Disease” OR “Neurologic Degenerative Conditions” OR “Degenerative Condition, Neurologic” OR “Degenerative Conditions, Neurologic” OR “Neurologic Degenerative Condition” OR “Neurologic Degenerative Diseases” OR “Degenerative Neurologic Diseases” OR “Degenerative Neurologic Disease” OR “Neurologic Disease, Degenerative” OR “Neurologic Diseases, Degenerative” OR “Degenerative Diseases, Central Nervous System” OR “Degenerative Diseases, Spinal Cord”) AND TS = (“artificial intelligence” OR “AI” OR “artificial-intelligence” OR “deep learning” OR “machine learning” OR “Intelligence, Artificial” OR “Computer Reasoning” OR “Reasoning, Computer” OR “Machine Intelligence” OR “Intelligence, Machine” OR “Computational Intelligence” OR “Intelligence, Computational”)). From the initial results, we selected publications written in English since the year 2000, specifying “Article” or “Review” as the document type. After data retrieval, we performed preliminary data processing using CiteSpace. Ultimately, 1,402 publications were included in the study, comprising 1,159 articles and 243 reviews. The entire process of literature retrieval and data downloading was completed on March 16, 2025.

### 2.2 Bibliometric and visualization analysis

This study employed three advanced bibliometric tools - VOSviewer v1.6.20, CiteSpace v6.4.R1, and Bibliometrix R package v4.3.2 - to conduct multidimensional analysis of research patterns in artificial intelligence in neurodegenerative diseases.

VOSviewer is a specialized bibliometric analysis tool designed to construct and visualize interactive network maps for identifying research patterns, collaborative relationships, and thematic clusters through co-occurrence, co-citation, or co-authorship analysis of entities such as keywords, authors, and publications (10, 11). Bibliometric network analyses were performed using VOSviewer, encompassing country/regional collaborative networks, institutional partnerships, journal publication patterns, co-citation relationships among periodicals, author cooperation clusters, and high-frequency keyword mapping, all of which were subsequently visualized through comprehensive graphical representations.

**Figure 2:**
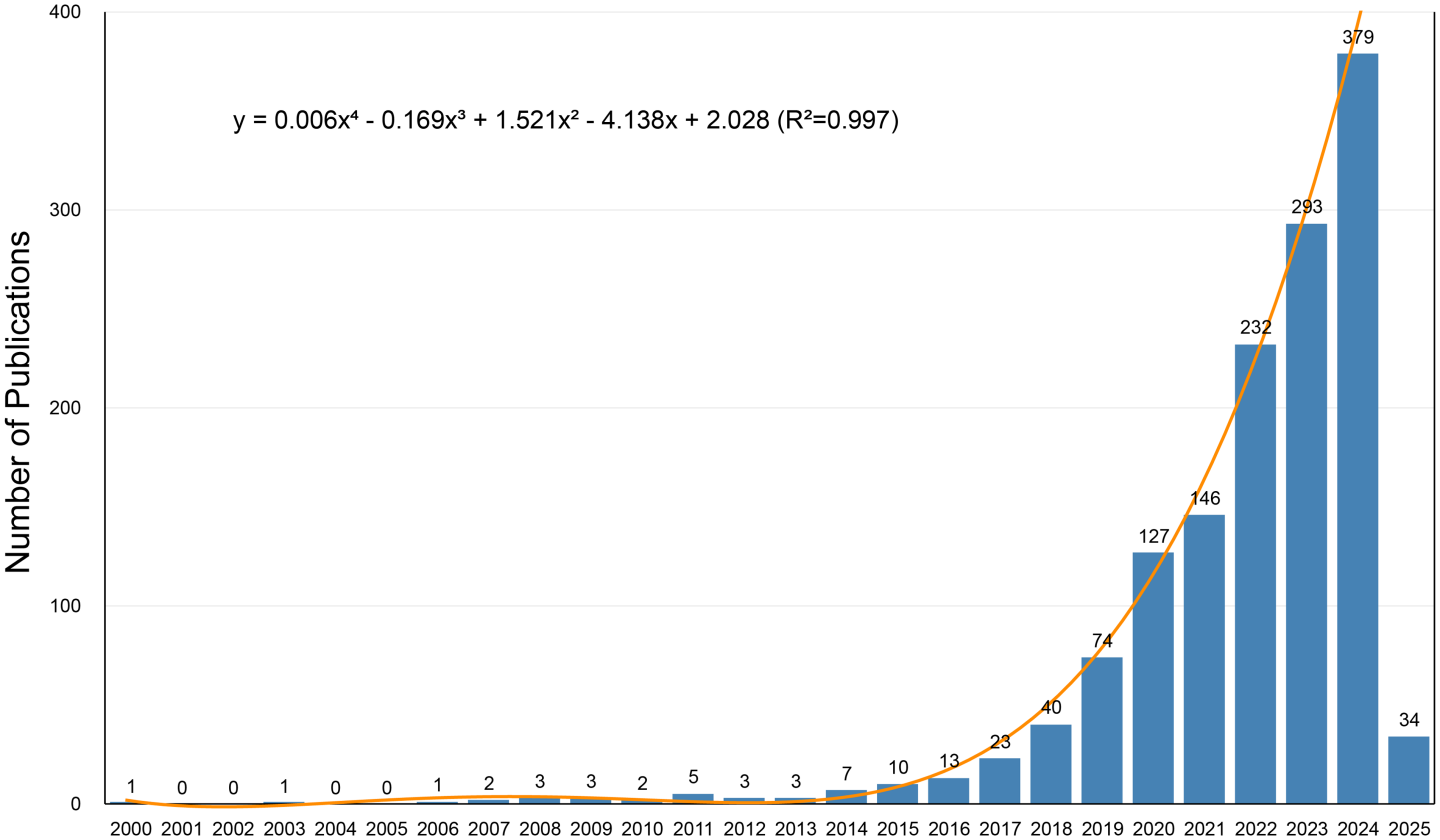
Annual publication trends over time.

CiteSpace excels in temporal analysis of scientific frontiers, identifying paradigm shifts and emerging trends through burst detection, time-slicing, and key pathway algorithms inspired by Kuhn’s theory of scientific revolutions (12–14). To investigate annual publication trends and identify critical research frontiers, CiteSpace was employed for multiple analytical dimensions: temporal distribution patterns of scholarly output, centrality metrics of national/regional contributions, burst detection of emerging terminology, and dual-map overlays of journal citation pathways - analyses which revealed both temporal patterns and spatial distributions in knowledge dissemination.

Bibliometrix offers comprehensive statistical and computational workflows in R, integrating metadata processing, citation analysis, and multi-dimensional visualization via its interactive Biblioshiny interface for reproducible bibliometric research (15–17). Through the Bibliometrix R package, core journals shaping this interdisciplinary domain were systematically identified, with their academic influence being quantitatively assessed through H-index and G-index (18, 19).

When analyzing countries and regions, we consolidated data by merging administrative divisions from the same nation (e.g., England, Scotland, Northern Ireland, and Wales into the United Kingdom) to ensure national-level consistency. For keyword analysis, we standardized synonyms to ensure conceptual consistency - for instance, unifying “Alzheimer disease, “and “AD” as “Alzheimer’s disease.” Two researchers independently verified the assessments. Any discrepancies were re-evaluated by a third-party researcher, and final consensus was reached through collaborative discussion among all three investigators.

## 3 Results

### 3.1 Basic quantitative information

A total of 1,402 relevant publications were retrieved from the SCI-EXPANDED database in WoSCC between January 1, 2000 and March 16, 2025. These records comprised 1,159 research articles and 243 review articles. Researchers from 86 countries/regions contributed to this field, involving 8,048 scholars affiliated with 2,637 institutions. The publications appeared in 509 academic journals and contained 3,315 author’s keywords. These works collectively cited 71,363 references from 12,374 distinct journal sources.

### 3.2 Annual publication trends

The annual publication trend provides a visual representation of the research field’s development (Figure 2). Since 2000, AI-related studies in neurodegenerative diseases have demonstrated a phased growth pattern. Annual publications remained consistently below 10 articles before 2014, with minor fluctuations but overall stagnation. A period of sustained growth began in 2014, transitioning to exponential growth after 2017. Publications reached 379 articles in 2024, with studies published since 2023 accounting for over half of the total output.

### 3.3 Analysis of countries and regions

**Figure 3:**
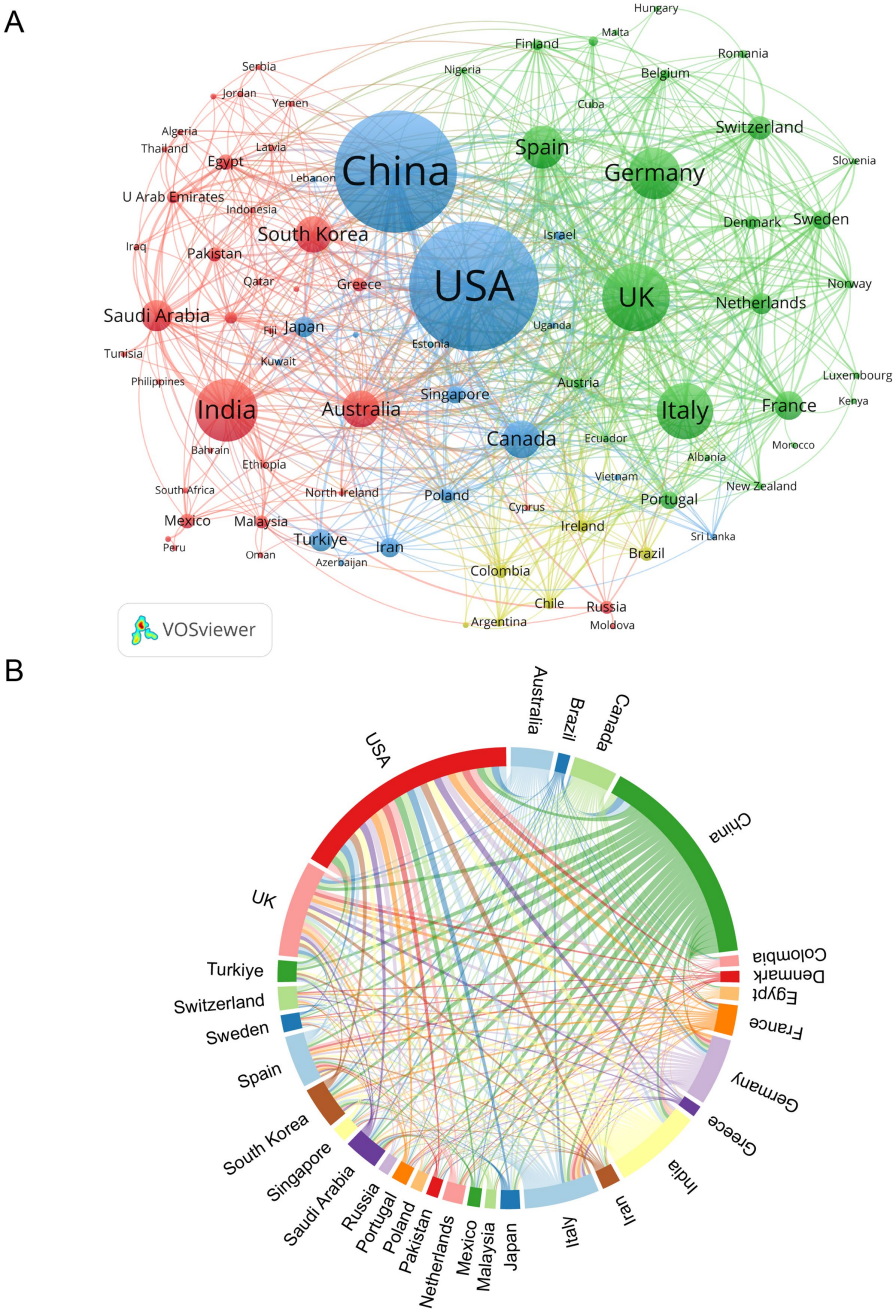
Analysis of countries/regions. **(A)** Academic collaboration networks between countries/regions. **(B)** Chord diagram illustrating country/region collaboration patterns.

As shown in Table 1 and Figure 3, the USA and China dominate in publication volume, contributing 25.96% (364 articles) and 24.11% (338 articles) of total outputs, respectively. The USA also leads in total citations (10,223), while the UK exhibits the highest citations per publication (31.68) and centrality (0.24), indicating strong international collaboration. India ranks fourth in publications (9.84%) but has the lowest citations per publication (13.62). Figure 3B highlights collaboration patterns among the top 30 countries, with the USA, China, and the UK forming central nodes in the network.

**Table 1:** Top 10 most productive countries and regions.

Rank	Country/Region	NP (%)	Citations	ACP	Centrality
1	USA	364 (25.96%)	10,223	28.09	0.17
2	China	338 (24.11%)	6,713	19.86	0.09
3	UK	152 (10.84%)	4,816	31.68	0.24
4	India	138 (9.84%)	1880	13.62	0.11
5	Italy	118 (8.42%)	2,664	22.58	0.1
6	Germany	106 (7.56%)	2,557	24.12	0.08
7	Spain	82 (5.85%)	1,467	17.89	0.06
8	Canada	69 (4.92%)	1939	28.10	0.15
9	Australia	67 (4.78%)	1776	26.51	0.17
10	South Korea	66 (4.71%)	1,675	25.38	0.03

NP, number of publications; ACP, average citations per publication.

### 3.4 Analysis of institutions

**Figure 4:**
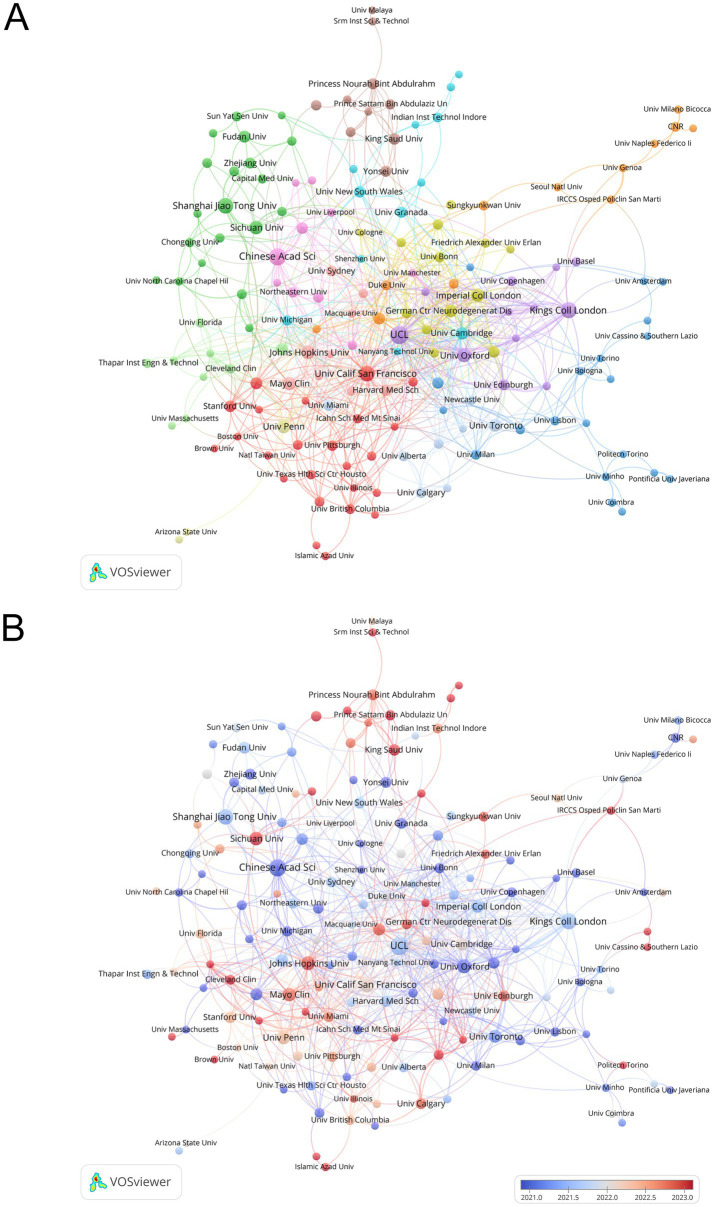
Analysis of institutions. **(A)** Map of the network of institutions with clusters. **(B)** Map of the network of institutions with average publication year.

As shown in Table 2, the top 10 productive institutions were dominated by the USA, China, and the UK, with UCL leading in publications and the Chinese Academy of Sciences ranking first in total citations. Notably, institutions from China, such as Sichuan University, exhibited the latest average publication year (2023.2), while the University of California San Francisco had the highest average citations per publication (42.4). Figure 4 illustrates the collaborative network and thematic clusters of institutions via a VOSviewer-generated map. Figure 4A highlights distinct clusters, with major institutions like UCL and University of California San Francisco forming central nodes, while Figure 4B reveals a temporal gradient in research output: institutions with earlier average publication years (pre-2021) appear in cooler blue tones, while those with more recent contributions (post-2022) trend toward warmer red hues, reflecting a “blue-gray-red” spectrum aligned with publication recency.

**Table 2:** Top 10 most productive institutions.

Rank	Institution	Publications	Citations	ACP	APY	Country
1	UCL	27	912	33.78	2021.56	USA
2	Chinese Acad Sci	24	1,358	56.58	2020.71	China
3	Kings Coll London	21	780	37.14	2021.52	UK
4	Shanghai Jiao Tong Univ	20	388	19.40	2021.45	China
5	Univ Calif San Francisco	20	848	42.40	2022.10	USA
6	Johns Hopkins Univ	16	344	21.50	2022.88	USA
7	Univ Oxford	16	391	24.44	2021.00	UK
8	Mayo Clin	15	273	18.20	2022.80	USA
9	Sichuan Univ	15	109	7.27	2023.20	China
10	Univ Penn	15	185	12.33	2022.33	USA

ACP, average citations per publication; APY, average publication year.

### 3.5 Analysis of journals and co-cited journals

**Figure 5:**
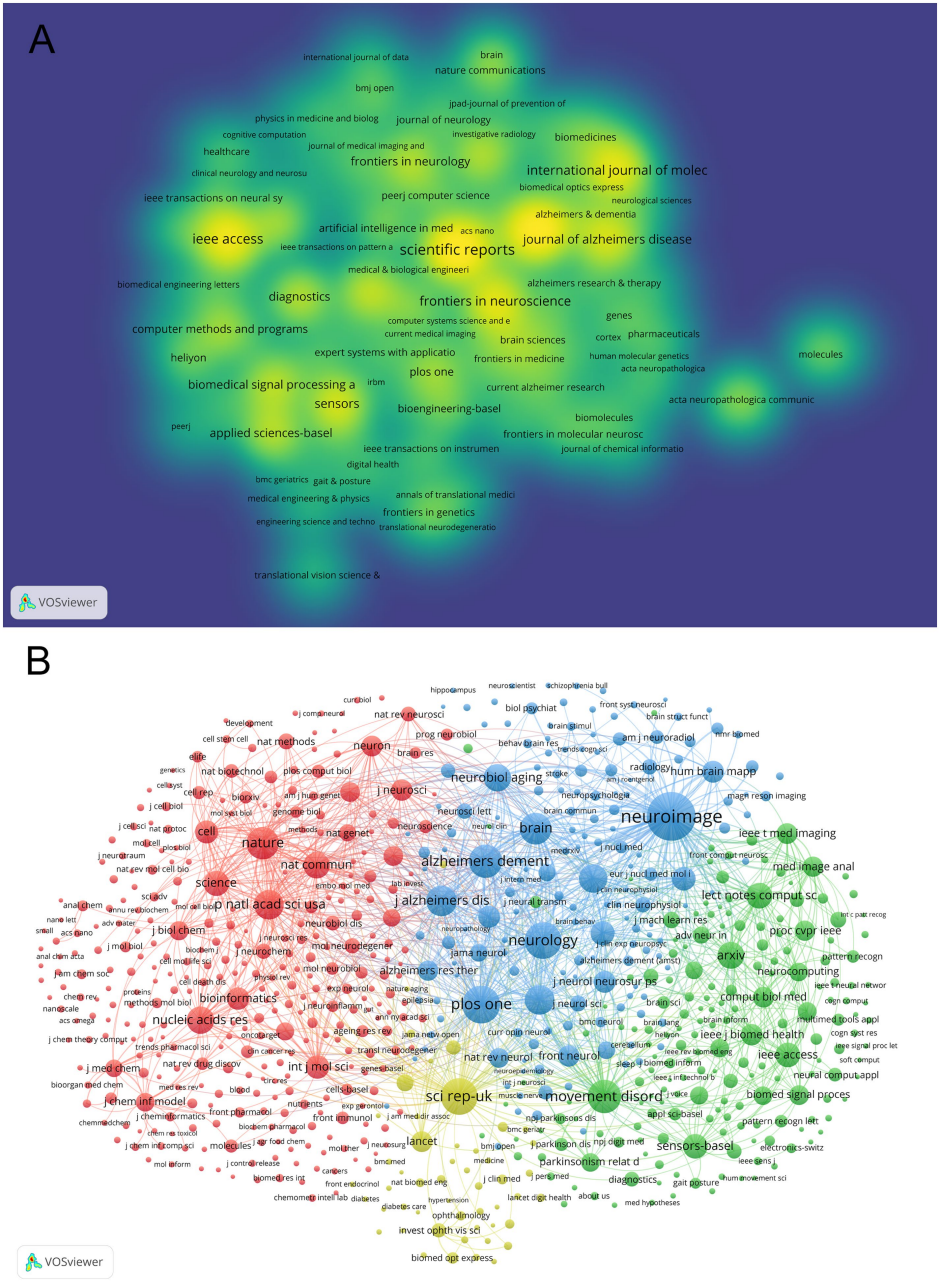
Analysis of journals and co-cited journals. **(A)** Co-occurrence network visualization of journals. **(B)** Co-cited network visualization of journals.

A total of 509 journals contributed to research in this field. Following Bradford’s Law of Scattering (20), 21 core journals were identified (Table 3). All these journals were classified as Q1/Q2 in the 2024 JCR rankings. As shown in Table 3 and Figure 5A, *Scientific Reports* ranked first with 47 publications, followed by *Frontiers in Aging Neuroscience* (46 publications) and *IEEE Access* (37 publications). Notably, *NeuroImage* achieved an exceptionally high citation rate (1,335 citations) despite its lower publication count (19 publications). As shown in Table 4 and visualized in Figure 5B, the journal co-citation analysis identified 12,374 journals, with *NeuroImage* (2,159 co-citations, IF = 4.7, Q1), *PLoS One* (1,299 co-citations, IF = 2.9, Q1), and *Neurology* (1,228 co-citations, IF = 8.4, Q1) as the top three most co-cited journals. High-impact journals such as *Nature* (940 co-citations, IF = 50.5, Q1) and *Science* (574 co-citations, IF = 50.5, Q1) were also prominent.

**Table 3:** The 21 core research productivity journals identified by Bradford’s law of scattering.

Rank	Journal	Publications	H-index	G-index	Citations	IF (2024)	JCR (2024)
1	Scientific Reports	47	14	27	813	3.8	Q1
2	Frontiers in Aging Neuroscience	46	15	25	717	4.1	Q2
3	IEEE Access	37	12	26	696	3.4	Q2
4	Frontiers in Neuroscience	30	12	26	726	3.2	Q2
5	International Journal of Molecular Sciences	29	9	18	352	4.9	Q1
6	Sensors	26	11	20	429	3.4	Q2
7	Computers in Biology And Medicine	23	11	23	538	7	Q1
8	Journal of Alzheimer’s Disease	23	6	13	185	3.4	Q2
9	Diagnostics	22	9	15	234	3	Q1
10	Applied Sciences-Basel	21	10	18	350	2.5	Q1
11	Biomedical Signal Processing and Control	21	5	10	121	4.9	Q1
12	Frontiers in Neurology	20	7	14	220	2.7	Q2
13	Neuroimage	19	11	19	1,335	4.7	Q1
14	IEEE Journal of Biomedical and Health Informatics	18	9	18	350	6.7	Q1
15	Computer Methods and Programs in Biomedicine	16	11	16	535	4.9	Q1
16	PLoS One	16	8	16	288	2.9	Q1
17	Neuroimage-Clinical	12	9	12	235	3.4	Q2
18	Artificial Intelligence in Medicine	11	7	11	201	6.1	Q1
19	Bioengineering-Basel	11	6	8	73	3.8	Q2
20	Heliyon	11	5	10	104	3.4	Q1
21	Biomedicines	10	5	9	90	3.9	Q1

**Table 4:** Top 10 co-cited journals.

Rank	Co-cited journal	Co-citations	IF (2024)	JCR (2024)
1	NeuroImage	2,159	4.7	Q1
2	PLoS One	1,299	2.9	Q1
3	Neurology	1,228	8.4	Q1
4	Scientific Reports	1,223	3.8	Q1
5	Movement Disorders	1,021	7.4	Q1
6	Alzheimers & Dementia	1,004	13.1	Q1
7	Nature	940	50.5	Q1
8	BRAIN	933	11.9	Q1
9	Journal of Alzheimer’s Disease	870	3.4	Q2
10	PNAS	806	9.4	Q1

#### 3.5.1 Dual-map overlay analysis of journals

**Figure 6:**
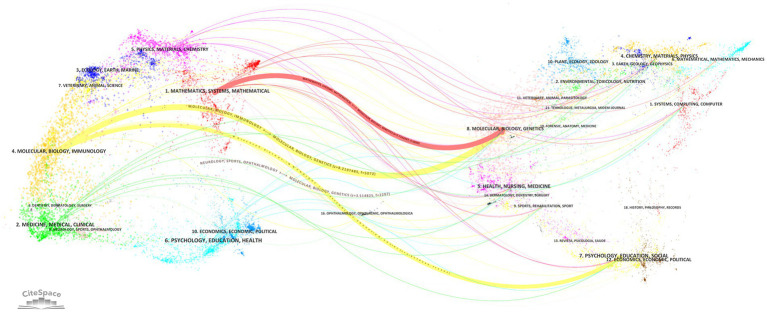
Dual-map overlay analysis of journals.

Developed by Chen and Leydesdorff, the dual-overlay analysis method for journals elucidates how citing and cited publications are spatially distributed across scientific domains (21). As illustrated in Figure 6, four thick lines and numerous thin lines depict citation relationships between journals. From top to bottom: The first thick red line extends from “MATHEMATICS, SYSTEMS, MATHEMATICAL” to “MOLECULAR, BIOLOGY, GENETICS” (*z* = 2.7162597, *f* = 1826). The second thick yellow line connects “MOLECULAR, BIOLOGY, IMMUNOLOGY” to “MOLECULAR, BIOLOGY, GENETICS” (*z* = 8.2197485, *f* = 5,072). The third thick yellow line links “MOLECULAR, BIOLOGY, IMMUNOLOGY” to “PSYCHOLOGY, EDUCATION, SOCIAL” (*z* = 1.8278345, *f* = 1,302). The fourth thick gray line spans from “NEUROLOGY, SPORTS, OPHTHALMOLOGY” to “MOLECULAR, BIOLOGY, GENETICS” (*z* = 3.514825, *f* = 2,297). These patterns collectively demonstrate the multidisciplinary nature of this research field.

### 3.6 Analysis of authors

**Figure 7:**
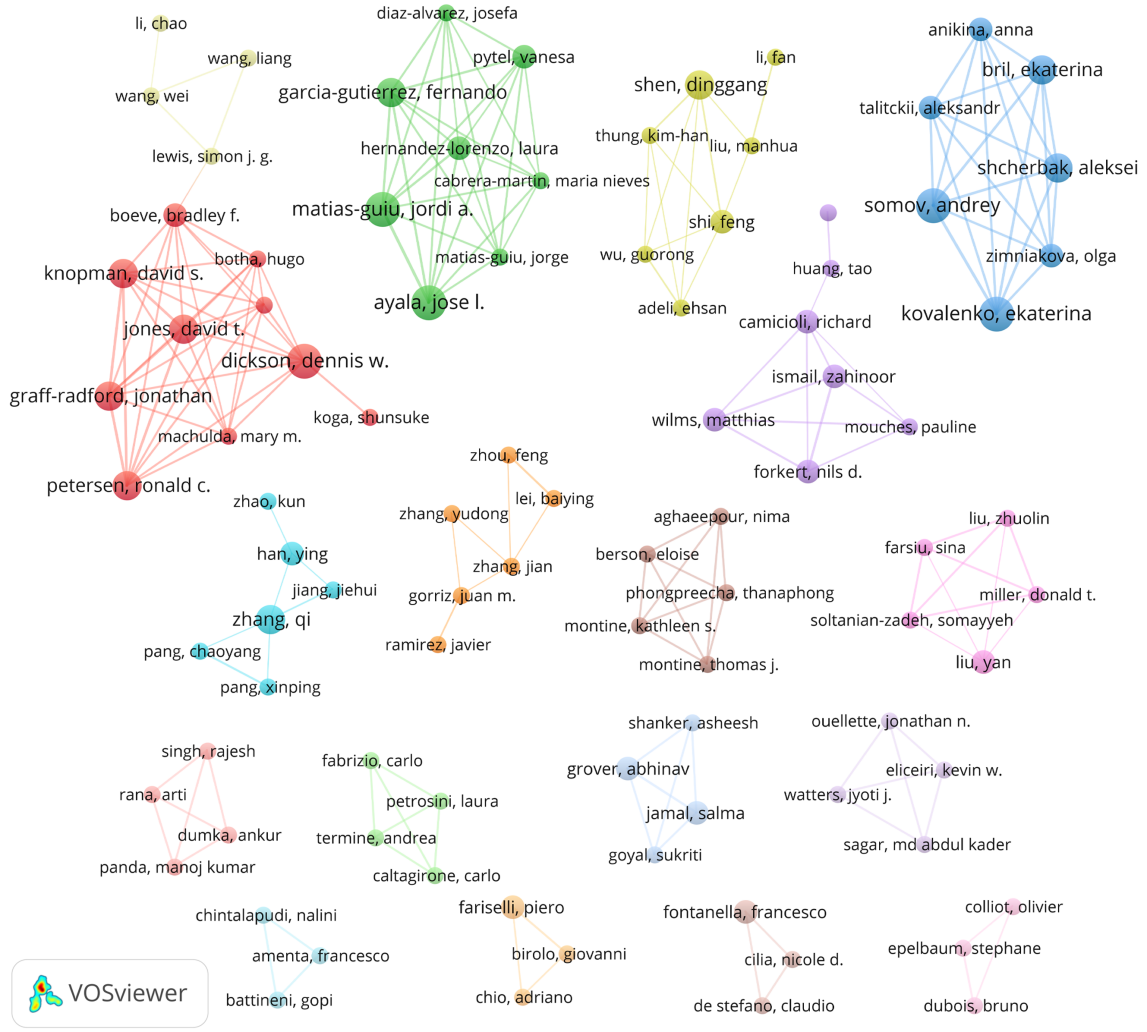
Author collaboration network.

A total of 8,048 authors contributed to this field. The most prolific authors, including Ayala, Matias-Guiu, Kovalenko, Somov and Dickson, each published 6 articles (Table 5). Notably, Dr. Shen achieved the highest total citations. As illustrated in Figure 7, prominent academic groups contributing to the field include: Ayala, Matias-Guiu et al. (green cluster); Kovalenko, Somov et al. (blue cluster); Dickson et al. (red cluster); and Shen et al. (yellow cluster). These distinct clusters, emphasize strong intra-group collaborations but limited inter-cluster connectivity, possibly reflecting thematic or institutional specialization.

**Table 5:** Top 10 most productive authors.

Rank	Author	Publications	Citations
1	Ayala, Jose L.	6	94
2	Matias-Guiu, Jordi A.	6	94
3	Kovalenko, Ekaterina	6	92
4	Somov, Andrey	6	92
5	Dickson, Dennis W.	6	54
6	Shen, Dinggang	5	624
7	Tanveer, M.	5	373
8	Graff-Radford, Jonathan	5	109
9	Jones, David T.	5	109
10	Knopman, David S.	5	109

### 3.7 Analysis of highly cited publications

Publications with high citations often represent critical milestones in the development of a research field. Table 6 lists information for the top 10 highly cited publications in the field of AI in neurodegenerative diseases.

**Table 6:** The top 10 cited publications.

Rank	Title	Year	Journal	First author	Total citations	DOI
1	The genetic architecture of Parkinson’s disease	2020	The Lancet Neurology	Blauwendraat, Cornelis	649	10.1016/S1474-4422(19)30287-X
2	Single subject prediction of brain disorders in neuroimaging: Promises and pitfalls	2017	NeuroImage	Arbabshirani, Mohammad R.	600	10.1016/j.neuroimage.2016.02.079
3	Clonally expanded CD8 T cells patrol the cerebrospinal fluid in Alzheimer’s disease	2020	Nature	Gate, David	555	10.1038/s41586-019-1895-7
4	Predicting Age Using Neuroimaging: Innovative Brain Ageing Biomarkers	2017	Trends in Neurosciences	Cole, James H.	519	10.1016/j.tins.2017.10.001
5	A research agenda for ageing in China in the 21st century (2nd edition): Focusing on basic and translational research, long-term care, policy and social networks	2020	Ageing Research Reviews	Fang, Evandro F.	352	10.1016/j.arr.2020.101174
6	Hierarchical Fully Convolutional Network for Joint Atrophy Localization and Alzheimer’s Disease Diagnosis Using Structural MRI	2020	IEEE Transactions on Pattern Analysis and Machine Intelligence	Lian, Chunfeng	340	10.1109/tpami.2018.2889096
7	Neuroimaging standards for research into small vessel disease-advances since 2013	2023	The Lancet Neurology	Duering, Marco	318	10.1016/s1474-4422(23)00131-x
8	Predictive markers for AD in a multi-modality framework: an analysis of MCI progression in the ADNI population	2011	NeuroImage	Hinrichs, Chris	308	10.1016/j.neuroimage.2010.10.081
9	Uncovering the heterogeneity and temporal complexity of neurodegenerative diseases with Subtype and Stage Inference	2018	Nature Communications	Young, Alexandra L.	307	10.1038/s41467-018-05892-0
10	Applications of machine learning to diagnosis and treatment of neurodegenerative diseases	2020	Nature Reviews Neurology	Myszczynska, Monika A.	289	10.1038/s41582-020-0377-8

Published in *The Lancet Neurology* in 2020, “The genetic architecture of Parkinson’s disease” ranks first with 649 citations. Blauwendraat et al. establishes a comprehensive genetic framework for Parkinson’s disease, enabling AI-driven approaches for risk prediction, patient stratification, and precision therapeutics by identifying 90 + risk loci and highlighting the need for machine learning to integrate genetic data with clinical phenotypes (22).

Published in *NeuroImage* in 2017, “Single subject prediction of brain disorders in neuroimaging: Promises and pitfalls” ranks second with 600 citations. This study by Arbabshirani et al. provides a comprehensive review of how to leverage artificial intelligence techniques to analyze multimodal neuroimaging data, enabling automated diagnosis, classification, and prediction of neurodegenerative diseases (23). It holds significant guiding importance for advancing the application of AI in the precision diagnosis and treatment of neurodegenerative diseases.

Published in Nature in 2020, “Clonally expanded CD8 T cells patrol the cerebrospinal fluid in Alzheimer’s disease” ranks third with 555 citations. This work provides critical antigen-specific immune signatures that can enhance AI-driven biomarker discovery and therapeutic target identification, demonstrating how computational integration of multi-omic data (mass cytometry, scRNA-seq, TCR sequencing, and machine learning) can decode neuroinflammation mechanisms in neurodegenerative diseases (24).

### 3.8 Burst analysis of references

**Figure 8:**
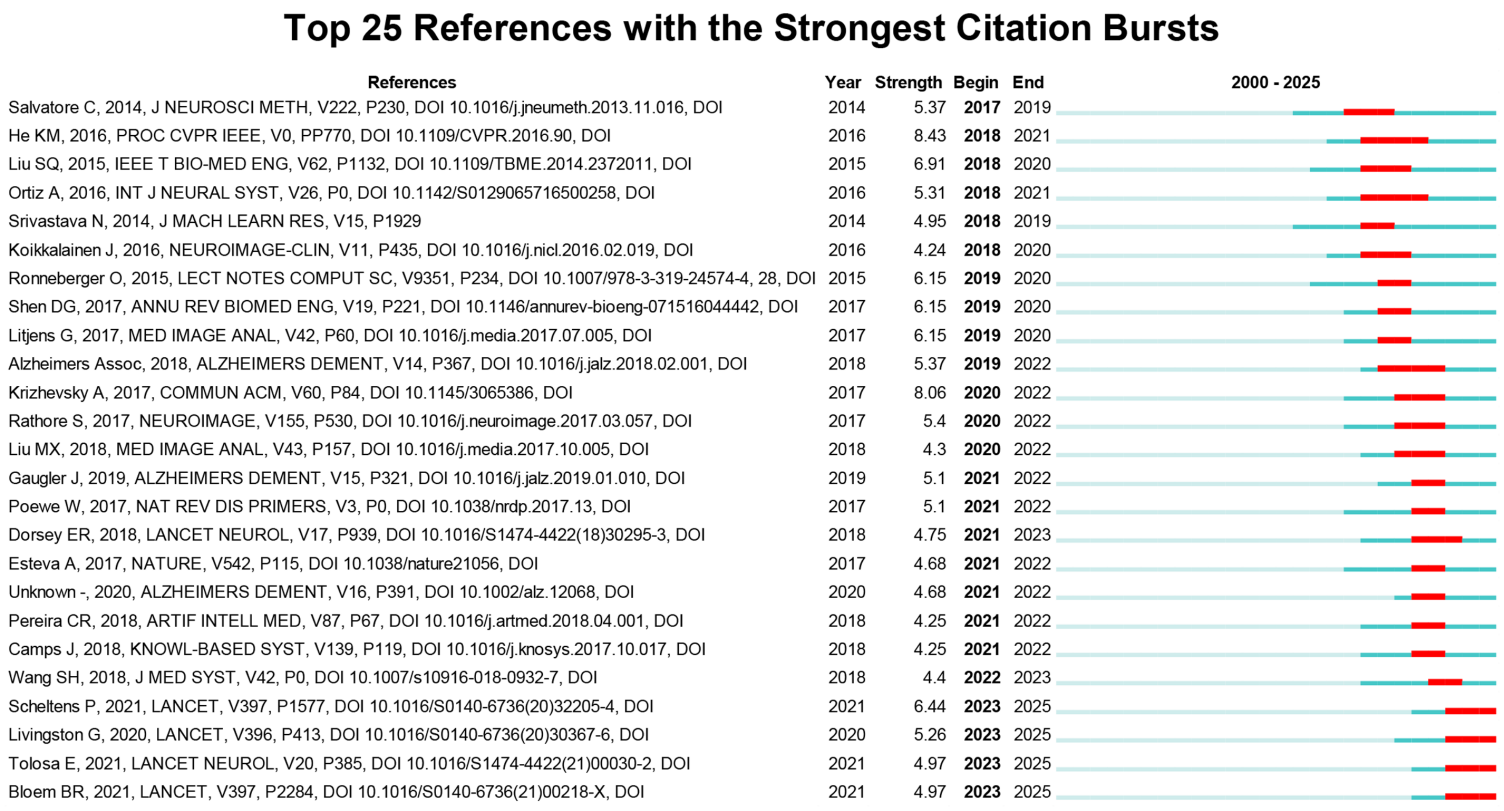
Top 25 references with the strongest citation bursts.

Burst analysis of references helps identify research that received considerable attention during different periods and pinpoint the time points when these citations surged. Figure 8 lists the top 25 co-cited references with the highest citation burst strength. Among these, the study by He et al. exhibited the most significant citation burst (Strength = 8.43, 2018–2021). Subsequent studies with high bursts include those by Krizhevsky et al. (Strength = 8.06, 2020–2022) and Liu et al. (Strength = 6.91, 2018–2020).

### 3.9 Analysis of keywords

#### 3.9.1 Keyword frequency

**Figure 9:**
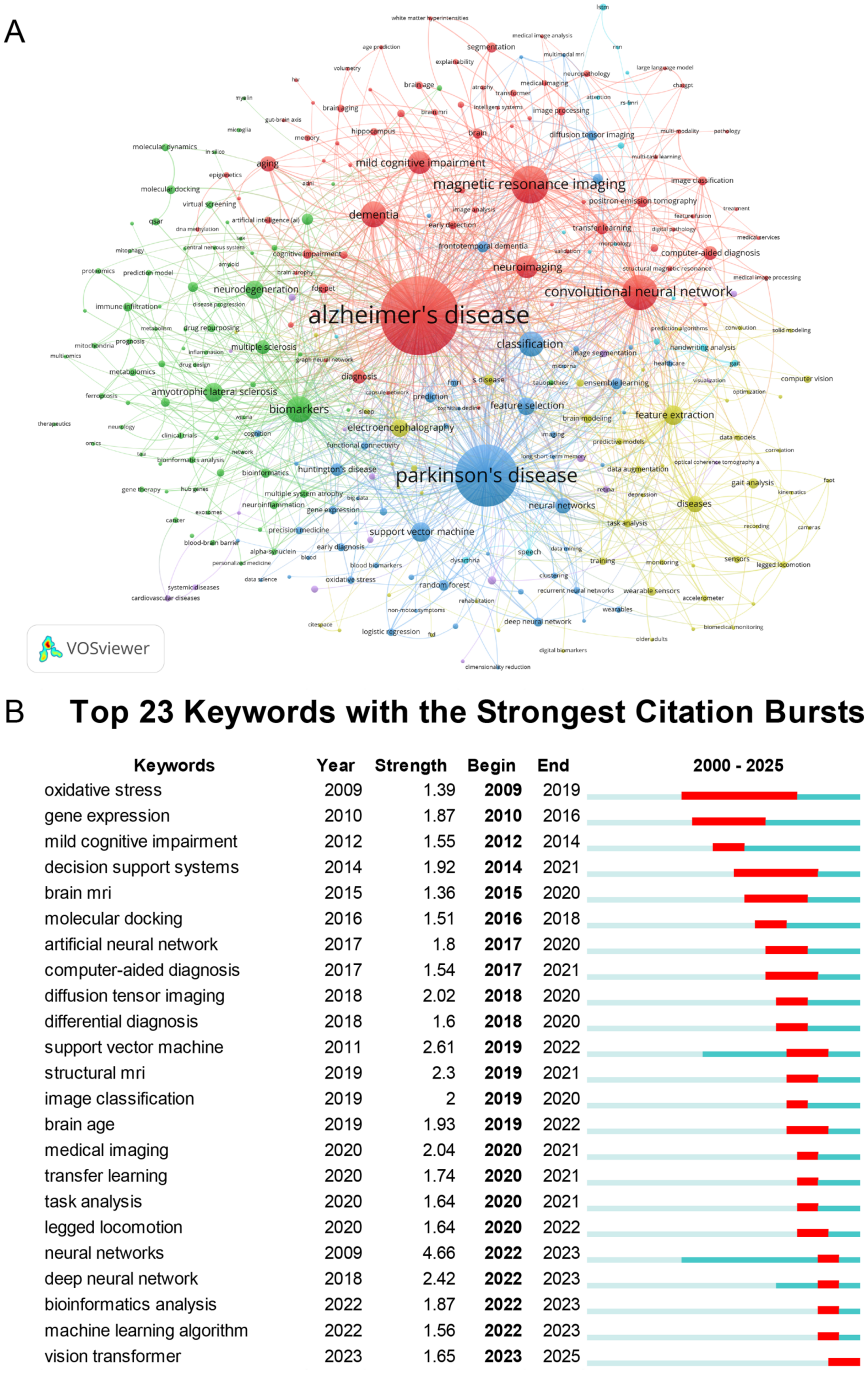
Analysis of keywords. **(A)** Keyword co-occurrence network. **(B)** Top 23 keywords with the strongest citation bursts.

Author keywords in publications are typically selected to emphasize core research themes and reflect study contents. Frequently occurring keywords represent key focuses within the research field. After excluding the search terms “Artificial Intelligence” and “Neurodegenerative Diseases,” we identified 287 keywords with occurrence frequencies ≥ 3 to establish a co-occurrence network (Figure 9A). As shown in Figure 9A, the top 10 high-frequency keywords were: “alzheimer’s disease,” “parkinson’s disease,” “magnetic resonance imaging,” “convolutional neural network,” “biomarkers,” “dementia,” “classification,” “mild cognitive impairment,” “neuroimaging,” and “feature extraction.”

#### 3.9.2 Keyword emergence analysis

Keyword emergence analysis identifies sudden surges in keyword frequency within specific timeframes pinpointing emerging research frontiers and reflecting real-time shifts in scientific focus within neurodegenerative diseases studies utilizing artificial intelligence. As shown in Figure 9B the top five keywords with the highest burst strength were: “neural networks” (strength = 4.66, 2022–2023) “support vector machine” (strength = 2.61, 2019–2022) “deep neural network” (strength = 2.42, 2022–2023) “structural MRI” (strength = 2.3, 2019–2021) and “medical imaging” (strength = 2.04, 2020–2021)

## 4 Discussion

### 4.1 General distribution

Based on the bibliometric analysis, this study systematically depicts the research landscape of AI in neurodegenerative diseases. Regarding publication trends, the exponential growth in publications since 2017 underscores the transformative impact of artificial intelligence in neurodegenerative diseases research. This surge aligns with advancements in deep learning architectures, increased computational power, and the availability of large-scale multimodal datasets, which have collectively enabled novel applications in disease prediction, biomarker discovery, and therapeutic development.

The prominence of journals like *Scientific Reports* and *Frontiers in Aging Neuroscience* is the interdisciplinary nature of this field, merging computational science with clinical neurology. The classification of all 21 core journals as Q1/Q2 in the 2024 JCR rankings confirms the high scientific rigor and impact of publications in this domain. Notably, *NeuroImage*’s exceptional citation rate despite fewer publications signals the critical role of neuroimaging data in AI-driven neurodegenerative research. The dual-map overlay analysis further reinforces the multidisciplinary integration, with strong citation pathways linking computational methodologies to molecular biology and clinical neurology.

### 4.2 Geographical analysis and leading academic teams

The dominance of the USA and China reflects their substantial investment in AI in neurodegenerative diseases research. However, the UK’s higher citations per publication and centrality suggest impactful contributions through international collaboration. India’s high output but low citations may indicate a focus on quantity over quality or limited global engagement. To address these disparities, developing countries need to strengthen international collaboration while actively improving research quality and global influence.

Institutions from the USA, China, and the UK dominated research productivity, highlighting their central roles in AI-driven neurodegenerative diseases studies. UCL (USA) emerged as the most prolific institution, underscoring its leadership in both output and influence within this interdisciplinary field.

Author collaborations demonstrate pronounced intra-group cohesion yet strikingly limited cross-cluster connectivity. This pattern likely stems from the field’s inherently interdisciplinary nature — medical researchers, computer scientists, and bioinformaticians predominantly operate within specialized disciplinary silos. The current collaboration framework reveals an urgent need for cross-disciplinary integration, particularly in bridging clinical expertise with computational innovation. Establishing structured interdisciplinary collaboration mechanisms could dismantle existing academic barriers, potentially catalyzing transformative breakthroughs in neurodegenerative diseases research.

### 4.3 Development history

By analyzing highly cited articles and reference burst analysis, we can identify research hotspots in different periods, thereby outlining the development trajectory of the AI in neurodegenerative disease research field. The development of this field has evolved through several key phases, marked by technological advancements and interdisciplinary collaborations. In 2011, Hinrichs et al. demonstrated the power of Multi-Kernel Learning to predict MCI-to-Alzheimer’s progression by fusing multi-modal biomarkers (imaging, cognitive, and biological data) into a unified Multi-Modality Disease Marker, highlighting machine learning’s potential to capture complex disease heterogeneity (25). In 2017, Arbabshirani et al. comprehensively review over 200 neuroimaging-based machine learning studies across various brain disorders, identify common methodological pitfalls, and propose solutions to enhance clinical applicability, thereby establishing a critical framework for improving AI-driven diagnosis and biomarker discovery in brain disorder research (23). Concurrently, Cole and Franke established “brain-predicted age” as a biomarker of brain health, demonstrating through machine learning that accelerated brain ageing (brain age > chronological age) correlates with advanced cognitive decline, increased risk of neurodegenerative diseases, and higher mortality (26). In 2018, Young et al. developed an AI method that identifies heterogeneous disease subtypes and their progression stages from cross-sectional data, significantly enhancing precision medicine in neurodegenerative disease research by improving patient stratification and outcome prediction (27). In 2020, Lian et al. propose a hierarchical fully convolutional network that jointly automates discriminative atrophy localization and Alzheimer’s disease diagnosis from structural MRI, significantly advancing AI in neurodegenerative disease research by enabling integrated (28). Myszczynska synthesized AI’s role in drug discovery and image interpretation, emphasizing its capacity to integrate high-dimensional data for actionable insights (29). In 2023, Duering updated the STRIVE neuroimaging standards (STRIVE-2) to define and harmonize MRI biomarkers of cerebral small vessel disease, emphasizing quantitative methods and emerging features like incidental DWI + lesions (30).

### 4.4 Hotspots and frontiers

The bibliometric analysis reveals four prominent research frontiers in AI in neurodegenerative diseases since 2000. Intelligent neuroimaging analysis represents a major research focus, demonstrated by strong citation bursts in “diffusion tensor imaging” (Strength = 2.02, 2018–2020) and “structural MRI” (Strength = 2.30, 2019–2021), alongside high co-occurrence frequencies for “magnetic resonance imaging” (118 occurrences) and “convolutional neural networks” (106 occurrences) with recent average publication years of 2022. Recent advancements in AI-driven neuroimaging have significantly facilitated the progress of neurodegenerative diseases research. Structural MRI and diffusion tensor imaging are pivotal for detecting early-stage brain atrophy, white matter integrity loss, and microstructural changes in conditions like Alzheimer’s disease and Parkinson’s disease (31, 32). Structural MRI enables precise quantification of region-specific atrophy (e.g., cerebral spinal fluid volume changes near the hippocampus in Alzheimer’s disease) via deep learning algorithms, enhancing diagnostic accuracy even with widely available 2D T1-weighted scans (33). AI-powered image classification models, particularly deep learning frameworks, enable high-accuracy differentiation of disease subtypes by integrating features from multimodal MRI data (34). Fixel-based analysis, reveals early white matter macrostructural changes (fiber cross-section reduction) specifically linked to tau pathology in Alzheimer’s disease, offering potential biomarkers for pre-symptomatic detection (35). Emerging frameworks also address MRI sequence recognition to standardize data preprocessing (36). Challenges remain in generalizing models across heterogeneous datasets, but emerging explainable AI frameworks address “black-box” limitations by providing interpretable saliency maps through gradient-weighted class activation mapping (36). Future research should prioritize multimodal integration and external validation across diverse cohorts to enhance clinical translation (37).

Machine learning methodological iterations show significant momentum through the burst of “neural networks” (Strength = 4.66, 2022–2023) and “deep neural networks” (Strength = 2.42, 2022–2023), further evidenced by “vision transformer” (Strength = 1.65, 2023–2025) and substantial co-occurrence of “transfer learning” (18 occurrences) and “explainable AI” (13 occurrences) in recent publications. The evolution of machine learning in neurodegenerative diseases research reflects a dynamic shift from traditional algorithms to advanced architectures. Early approaches, such as support vector machines (SVMs), demonstrated utility in biomarker prediction and early disease classification by leveraging handcrafted features from neuroimaging or cerebrospinal fluid data (38). However, their reliance on handcrafted features limits scalability. Artificial neural networks (ANNs), including multilayer perceptrons, improved drug delivery predictions by integrating perturbation theory and machine learning (IFPTML-ANN models) (39). The rise of deep neural networks (DNNs), especially convolutional neural networks (CNNs), revolutionized neuroimaging analysis. For instance, 3D-CNN outperformed many models in Alzheimer’s disease detection by leveraging spatial patterns from MRI data, achieving the highest accuracy in multi-class classification tasks (40). Emerging vision transformers (ViTs) leverage self-attention mechanisms to process global contextual features in medical imaging (41). ViTs outperform CNNs in MRI-based Alzheimer’s Disease classification when trained on limited datasets, achieving nearly fourfold improvements in accuracy and computational efficiency (42). This progression underscores a paradigm shift from manual feature engineering (SVM) to automated, context-aware learning (ViTs/DNNs), driven by neuroimaging and multimodal data integration (43, 44). Future hotspots include hybrid architectures (45, 46) and interpretable AI to bridge clinical translation gaps (47).

Molecular mechanisms and drug discovery maintain sustained interest, reflected in “molecular docking” burst activity (Strength = 1.51, 2016–2018) and current high co-occurrence of “drug discovery” (13 occurrences, avg. pub. year 2022.15) and “bioinformatics” (12 occurrences, avg. pub. year 2022.08), complemented by recent “biomarker” research prominence (67 occurrences). Recent advances in AI-driven research have significantly enhanced the understanding of neurodegenerative diseases pathogenesis and therapeutic development. Molecular docking combined with AI approaches has been applied to expedite virtual screening. Computational tools and deep learning methods assist in identifying dual-target ligands (e.g., A2A receptor antagonists and MAO-B inhibitors) for Parkinson’s disease, as demonstrated in recent studies (48). A recent study combined docking with molecular dynamics simulations, including binding pose metadynamics, to identify a novel JNK3 inhibitor, which demonstrated efficacy in reducing TNF-*α* release and holds potential for mitigating neuroinflammation (49). Bioinformatics pipelines leverage AI and machine learning approaches to decode neurodegenerative disease-specific pathways, employing network analysis tools such as WGCNA and STRING. These methods have been successfully applied to investigate network analysis of neurodegenerative disease-associated genes, such as those linked to α-synuclein interactions in Parkinson’s disease (50). Multi-omics integration facilitates biomarker discovery, with hiPSC-derived 3D models enabling the identification of cell-type-specific drug responses (51). These approaches synergistically bridge molecular insights and AI-driven drug design.

Clinical decision support systems exhibit robust development patterns through extended citation bursts in “decision support systems” (Strength = 1.92, 2014–2021) and “computer-aided diagnosis” (Strength = 1.54, 2017–2021), with emerging “diagnostic model” co-occurrences (7 occurrences, avg. pub. year 2023) signaling ongoing innovation in clinical translation. Recent advancements in AI-driven clinical decision support systems (CDSS) for neurodegenerative diseases focus on enhancing diagnosis accuracy. For Alzheimer’s disease, novelty detection models trained on healthy control data, combined with a distance-to-boundary strategy, enable early risk identification by detecting deviations from normal cognitive patterns (52). Knowledge-based systems leveraging decision tables and semantic networks have shown efficacy in multiple sclerosis diagnosis, achieving 99% accuracy in trials by analyzing 45 clinical parameters (53). Moreover, ensemble learning optimizes differential diagnosis by fusing structural/functional connectivity data, as demonstrated in Parkinson’s disease and amyotrophic lateral sclerosis classification studies (54). Similarly, in Parkinson’s disease, mHealth-based CDSS integrate wearable sensor data, patient-reported symptoms, and clinical assessments to optimize treatment plans amid symptom complexity and disease fluctuation (55). Challenges persist, including heterogeneous data integration and technical uncertainty. For example, mHealth-based CDSS for Parkinson’s disease faces “technical uncertainty” due to variable patient-reported and sensor-derived data, complicating real-world implementation (55). Emerging trends focus on low-cost, non-invasive tools and multimodal integration of clinically accessible data (e.g., gait, speech, handwriting), aiming to enable early neurodegenerative diseases detection in clinical and potentially home settings (56). Overall, AI-powered CDSS are pivotal in advancing precision diagnostics, yet require robust clinical validation and interdisciplinary collaboration to bridge implementation gaps.

### 4.5 Future directions

Through analyzing recent high-frequency keywords from VOSviewer co-occurrence mapping and CiteSpace burst detection, the future directions in AI for neurodegenerative disease research include the following aspects.

Advanced deep learning models may be a key future direction, as evidenced by the strong burst strength of “neural networks” (Strength 4.66, 2022–2023) and “deep neural network” (Strength 2.42, 2022–2023) in CiteSpace, alongside high occurrence and recent average publication years in VOSviewer data, such as “convolutional neural network” (106 occurrences, avg. year 2022.10) and “vision transformer” (6 occurrences, avg. year 2023.83), indicating growing adoption for tasks like image classification and disease diagnosis.

Integration of bioinformatics and multi-omics approaches may be a major focus, supported by the citation burst of “bioinformatics analysis” (Strength 1.87, 2022–2023) and high-frequency keywords like “drug repurposing” (10 occurrences, avg. year 2023) and “multi-omics” (3 occurrences, avg. year 2022) in the VOSviewer map, reflecting trends toward combining genetic, proteomic, and AI-driven analyses for uncovering molecular pathways in diseases like Alzheimer’s.

Explainable AI for clinical decision support may emerge as a critical area, with VOSviewer showing recent prominence of “explainable artificial intelligence” (13 occurrences, avg. year 2023.23) and “diagnostic model” (7 occurrences, avg. year 2023), complemented by burst analysis keywords such as “decision support systems” (Strength 1.92, 2014–2021) and “computer-aided diagnosis” (Strength 1.54, 2017–2021), suggesting a shift toward transparent AI systems for reliable diagnostics and treatment planning.

Early detection using digital biomarkers and multimodal data may be a significant direction, as indicated by high-frequency VOSviewer terms like “early detection” (10 occurrences, avg. year 2022.5) and “wearable sensors” (11 occurrences, avg. year 2022.36), along with burst keywords such as “mild cognitive impairment” (Strength 1.55, 2012–2014) and “gait analysis” emerging in recent data, highlighting the role of AI in non-invasive monitoring and predictive modeling for early intervention.

Emerging technologies like transformers and telemedicine may drive innovation, evidenced by the strong burst of “vision transformer” (Strength 1.65, 2023–2025) and “large language model” (3 occurrences, avg. year 2024) in the datasets, alongside keywords such as “telemedicine” (7 occurrences, avg. year 2021.29) and “wearables” (6 occurrences, avg. year 2022.33), pointing to AI advancements in remote healthcare, personalized medicine, and real-time disease management.

## 5 Limitation

While this bibliometric analysis provides a comprehensive overview of artificial intelligence in neurodegenerative diseases research, several limitations should be acknowledged. First, the exclusive reliance on the WoSCC database may have introduced selection bias, as relevant studies indexed in other databases were excluded. Second, the restriction to English-language publications and article/review formats likely underrepresented contributions from non-English-speaking regions and emerging research disseminated through conference proceedings or gray literature. Third, keyword standardization, though rigorously performed, may have inadvertently omitted nuanced terminology due to evolving AI subfield lexicons. Finally, the rapid pace of AI innovation may not be fully captured due to the inherent citation lag in scholarly publishing.

## 6 Conclusion

This bibliometric analysis reveals rapid growth in AI in neurodegenerative diseases research since 2017. The USA and China lead in productivity, while the UK excels in collaborative influence. Highly cited publications and burst references highlight important milestones in the development history. Key hotspots include intelligent neuroimaging analysis, machine learning methodological iterations, molecular mechanisms and drug discovery, and clinical decision support systems for early diagnosis. Future priorities include: advanced deep learning models, integration of bioinformatics and multi-omics approaches, explainable AI for clinical decision support, early detection using digital biomarkers and multimodal data, emerging technologies like transformers and telemedicine.
